# Inhibition of retinoic acid receptor α phosphorylation represses the progression of triple-negative breast cancer via transactivating miR-3074-5p to target DHRS3

**DOI:** 10.1186/s13046-021-01941-7

**Published:** 2021-04-26

**Authors:** Siyue Lou, Hang Gao, Huanwu Hong, Zhihui Zhu, Huajun Zhao

**Affiliations:** School of Pharmaceutical Sciences, Zhejiang Chinese Medical University, #548 Binwen Road, Hangzhou, 310053 China

**Keywords:** RARα, Triple-negative breast cancer, Phosphorylation, miR-3074-5p, DHRS3

## Abstract

**Background:**

Retinoids are promising agents in the treatment of different types of neoplasia including estrogen receptor-positive breast cancers, whereas refractoriness/low sensitivity is observed in triple-negative breast cancer (TNBC) subtype. However, the reason for these diverse retinoid-sensitivity remains elusive.

**Methods:**

Determinants of retinoid sensitivity were investigated using immunohistochemistry of primary patient samples, and identified retinoic acid receptor α (RARα) as a putative factor. The anti-tumor activity of hypo-phosphorylated RARα was investigated in TNBC cell models and a xenograft mouse model. Next, miRNA sequencing analysis was performed to identify the target miRNA of RARα, and luciferase reporter was used to confirm the direct target gene of miR-3074-5p.

**Results:**

We discovered that serine-77 residue of RARα was constantly phosphorylated, which correlated with TNBC’s resistance to retinoids. Overexpression of a phosphorylation-defective mutant RARαS77A mimicked activated RARα and repressed TNBC cell progression both in vitro and in vivo*,* via activating cell cycle arrest, apoptosis, and cytotoxic autophagy, independent of RARα agonists. We further revealed that the anti-tumor action of RARαS77A was, at least in part, mediated by the up-regulation of miR-3074-5p, which directly targeted *DHRS3*, a reductase negatively associated with TNBC patient survival. Our results suggest that the inhibition of RARαS77 phosphorylation by either expressing RARαS77A or inhibiting RARα’s phosphokinase CDK7, can bypass RA stimuli to transactivate tumor-suppressive miR-3074-5p and reduce oncogenic DHRS3, thus overcoming the RA-resistance of TNBC.

**Conclusion:**

The novel regulatory network, involving RARαS77 phosphorylation, miR-3074-5p, and DHRS3, emerges as a new target for TNBC treatment.

**Supplementary Information:**

The online version contains supplementary material available at 10.1186/s13046-021-01941-7.

## Background

Triple-negative breast cancer (TNBC) is a heterogeneous disease characterized by a lack of estrogen receptor (ER), progesterone receptor (PR), and HER2, and comprise approximately 15–20% of breast cancers [1]. They represent a challenge clinically due to the lack of targeted therapies coupled with an aggressive disease course, leaving cytotoxic chemotherapy and radiotherapy as the mainstay of treatment [2], thus driving research to find better therapeutics to improve the outcomes for this subtype.

All-trans retinoic acid (ATRA or RA) is the most potent natural form of vitamin A. It inhibits proliferation and induces differentiation/apoptosis in a variety of cancer cells and holds great promise as a chemotherapeutic agent [3–5], especially in the treatment of acute promyelocytic leukemia (APL) [6–8]. However, in breast carcinoma cells, only luminal and ER+ subtype are considered sensitive to retinoids [9, 10], while the majority of TNBC are unresponsive to RA treatment [10]. Therefore, comprehensive studies into the mechanism of RA-resistance may find alternative ways to bypass RA stimuli and offer novel therapies to treat these refractory breast cancers.

The canonical action of RA is exerted by its binding and activating nuclear retinoic acid receptors RARα, β, and γ. RAR dimerizes with retinoid-X-receptor (RXR) and regulates transcription of genes with retinoic acid response element (RARE) sequences in their promoters [3–5]. Among RAR subunits, RARα is the principal mediator of RA responsiveness in breast cancer [10]. As a phosphoprotein, RARα is a substrate of the cyclin-dependent kinase-activating kinase (CAK) complex consisting of CDK7 [11], cyclin H [12], and MAT1 [13, 14]. Ser-77 located at the AF-1 domain of RARα (RARαS77) is the main residue phosphorylated by CAK [15, 16]. Previous studies have shown that hyper-phosphorylation of RARα by CAK is associated with increased proliferation of acute myeloid leukemia (AML) and other types of cancer cells, whereas RA-induced RARα hypo-phosphorylation or mimicked hypo-phosphorylation by expressing RARαS77A mutant inhibited proliferation of cancer cells [17–21]. Of note, when expressing RARα and RARαS77A in parallel in RA-resistant AML cell line harboring a defective RARα ligand binding domain or in embryonic teratocarcinoma RARα^−/−^ stem cells, RARαS77A, but not RARα, inhibited proliferation without a need of RA stimulation [21]. These findings suggest that hypo-phosphorylated RARαS77 may overcome RA-resistance and functions as an activated form of RARα. However, the correlation between phosphorylation level of RARαS77 and RA-resistance, as well as the effects of RARαS77A in breast cancer cells, remains unknown.

MicroRNAs (miRNAs) are a class of endogenous small noncoding RNAs that regulate gene expression post-transcriptionally by binding to their target mRNAs for degradation and/or translational repression [22]. Dysregulated miRNAs are involved in several cellular processes of TNBC, exerting their function as either oncogenes or tumor suppressor genes [23]. Recent evidence indicates that several miRNAs can be modulated by nuclear receptors such as RARα [24]. By binding to RARE regions of target genes, RARα can regulate miR-10a [25] and miR-21 [9] transcription to participate in proliferation and motility of tumor cells. However, the function and role of miRNAs in TNBC are still not fully understood, and no study has demonstrated whether hypo-phosphorylated RARαS77 is equally capable of regulating transcription of miRNA as that of RARα.

We show here that RARαS77 hyper-phosphorylation contributes to RA-resistance and tumor progression of TNBC by transcriptional suppression of miR-3074-5p, thus suggesting the RARα/miR-3074-5p/DHRS3 axis may serve as novel therapeutic targets for TNBC.

## Materials and methods

### Chemicals and reagents

DMSO, DAPI, and MTT were purchased from Sigma-Aldrich (St. Louis, MO, USA). Propidium iodide (PI)/RNase staining kit and Annexin V-APC/7-AAD kit were purchased from Becton Dickinson (San Diego, CA, USA). The TUNEL kit was purchased from Yeasen Biotech (Shanghai, China). 3-MA was purchased from Selleckchem (Houston, TX, USA). ATRA, AM80, AM580, and THZ-1 were purchased from MedChemExpress (Monmouth Junction, NJ, USA). Antibody information is detailed in Supplemental Methods.

### Cell lines and cell culture

Human breast cancer cell lines were purchased from the cell bank of Shanghai Institute of Materia Medica, Chinese Academy of Sciences (Shanghai, China). Human embryonic kidney cells 293FT cells were purchased from National Infrastructure of Cell Line Resource (Beijing, China). TNBC cells were cultured in L15 medium, whereas other breast cancer cells were cultured in DMEM medium. 293FT cells were cultured in DMEM-high glucose medium (Gibco, NY, USA). All culture medium was supplemented with 10% fetal bovine serum (Gibco), penicillin (100 U/mL), and streptomycin (100 μg/mL).

### Primary specimens and tissue microarrays

Tumorous and their adjacent non-tumorous TNBC tissues were collected from 10 patients who underwent surgery at Zhejiang Cancer Hospital. Written informed consent was obtained from each patient and the study protocol conformed to the ethical guidelines of the 1975 Declaration of Helsinki and was approved by the Institute Research Ethics Committee of the Zhejiang Cancer Hospital. Details of tissue microarrays (TMA) immune-staining and scoring methods are given in the Supplemental Methods.

### Immunohistochemistry (IHC) analysis

Immunohistochemistry (IHC) analysis was performed as described [26]. Further details are given in the Supplemental Methods.

### Plasmid construction, transfection, and lentiviral production

The pcDNA3.0-RARα and pLVX-AcGFP-N1-RARα plasmids were purchased from Shanghai Nuoyue Biotechnology Co., Ltd. The p-Enter-DHRS3 plasmid was purchased from Vigene biosciences (Shandong, China). The pcDNA3.0-RARαS77A, pLVX-AcGFP-N1-RARαS77A, and p-Enter-DHRS3-Y188H were constructed using QuickMutation™ Plus gene site-directed mutation Kit (Beyotime, Shanghai, China). Plasmid transfection and lentiviral production were performed as described [20], with minor modifications detailed in the Supplemental Methods.

### Colony formation and cell viability assay

Colony formation and cell viability assay were performed as described previously [27]. Details are given in the Supplemental Methods.

### Western blotting (WB), cell cycle, and cell apoptosis analysis

WB, cell cycle, and apoptosis analysis were performed as described [27], and detailed in the Supplemental Methods.

### Acridine orange (AO) staining

See details in Supplemental Methods.

### In vivo animal studies

MDA-MB-231 cells (2 × 10^6^ cells, suspended in 0.1 mL PBS) overexpressing either empty vector, RARαS77A, or RARα were injected subcutaneously into 4-week-old BALB/c nu/nu female mice (Shanghai Experimental Animal Center, Shanghai, China). Tumor growth was measured every 3 days and tumor volume was calculated according to the formula: 1/2 × length × width^2^. After 26 days, the mice were sacrificed and the xenografts were removed for TUNEL staining and IHC analysis. All animal experiments were reviewed and approved by the Institutional Animal Care and Use Committee.

### TUNEL staining

Briefly, tumor tissues were fixed in 4% paraformaldehyde, cut into 5 μm sections, and stained as the manufacture’s instruction. Then, the sections were immersed into DAPI staining. TUNEL-positive (red) and DAPI-positive (blue) staining patterns were acquired under a fluorescence microscope (Nikon, Japan).

### Expression profile analysis of miRNAs

Total RNAs were extracted from MDA-MB-231 blank cells and RARαS77A-overexpressing cells using Trizol reagent (Invitrogen, Carlsbad, CA). Comprehensive miRNA expression analysis was performed using a NEBNext® Multiplex Small RNA Library Prep Set for Illumina® and an Illumina Hiseq 2500 platform (Novogene Bioinformatics Technology Co. Ltd., Beijing, China), which detects mature miRNAs. The data has been deposited in GEO: GSE160295.

### RNA isolation and quantitative reverse-transcriptase PCR (qRT-PCR)

RNA isolation and qRT-PCR analysis were performed as described [20]. The primers for targeted genes and miRNA were listed in the Supplemental Methods.

### miRNA mimics

miR-3074-5p mimic/mimic-NC were purchased from Ribo Bio (Guangzhou, China). The sense and antisense sequence of miR-3074-5p mimic were GUUCCUGCUGAACUGAGCCAG and CUGGCUCAGUUCAGCAGGAAC. The sense and antisense sequence of mimic-NC were UUUGUACUACACAAAAGUACUG and CAGUACUUUUGUGUAGUACAAA. The transfection system was the mixture of 1 × riboFECTTMCP Buffer, 100 ng/μl riboFECTTMCP Regent, and mimic (100 nM), and the mixture was added drop-wise to the appropriate wells, respectively. Then, the medium with miRNA transfection was changed after 4–6 h.

### Luciferase reporter assay

2 × 10^5^ 293FT cells were plated in 24-well for 24 h, followed by transfection with mimic-NC or mimic-miR-3074-5p, pmirGLO, wild type pmirGLO-DHRS3 3′-UTR. Transfection was performed using Lipofectamine 2000 (Invitrogen, Shanghai, China) based on the manufacturer’s protocol. The activities of luciferase were normalized to firefly luciferase.

### Statistical analysis

All data are expressed as mean ± SD. Statistical significance was analyzed using the Student’s t-test. The criterion of statistical significance was *p < 0.05; ** *p* < 0.01; ***p < 0.001.

## Results

### Constant phosphorylation of RARαS77 in human TNBC cells associates with RA-resistance

**Fig. 1:**
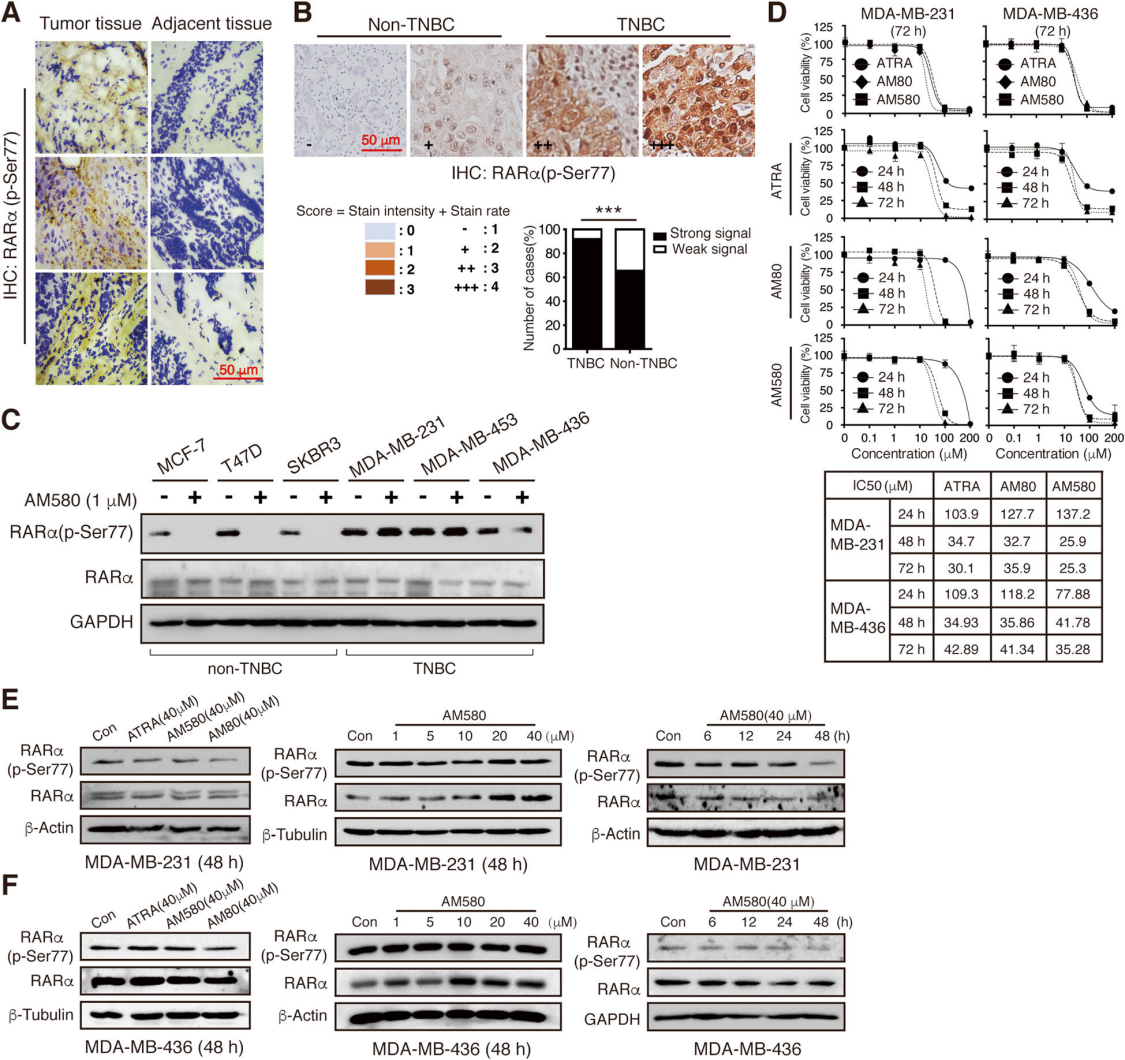
Constant phosphorylation of RARαS77 in human TNBC cells associates with RA-resistance. **a** Representative picture of RARα (p-Ser77) expression in human TNBC tissues and the neighboring non-tumorous tissues by immunohistochemical (IHC) staining. Scale bar, 50 μm. **b** Representative staining intensity of breast tumor tissue microarrays classified as four different grades. IHC score is the sum of staining intensity and positive cell rate. Scale bar, 50 μm. TNBC strong signal vs. non-TNBC strong signal, *** p < 0.001. **c** Western blotting analysis of RARαS77 phosphorylation levels in non-TNBC cell lines (MCF-7, T47D, and SKBR3) and TNBC cell lines (MDA-MB-231, MDA-MB-453 and MDA-MB-436) with or without RARα agonist AM580 for 48 h. **d** MDA-MB-231 and MDA-MB-436 cells were treated with pan-RARs agonist ATRA, RARα/β agonist AM80, and RARα agonist AM580 for indicated concentrations and time. Cell viability was assessed by MTT analysis and IC_50_ values were calculated. After treatment of different RARs agonists for indicated concentrations and time, RARα (p-Ser77) and total RARα expression levels were detected by western blotting in (**e**) MDA-MB-231 and (**f**) MDA-MB-436 cells

To evaluate the phosphorylation level of RARαS77 in TNBC, immunohistochemistry (IHC) was performed to assess the expression of RARα (p-Ser77) protein in a set of 10 tumors and adjacent non-tumorous TNBC tissues. The expression of RARα (p-Ser77) was significantly higher in TNBC specimens versus the non-tumorous tissues (Fig. 1a), thus suggesting a plausible role of RARαS77 phosphorylation in TNBC cells. Due to the limited number of patient samples, in order to verify if the above findings were consistent with a different analysis platform and patient cohort, we performed IHC analyses using human breast cancer tissue microarray (TMA) slides. Similar to the data obtained with the patient samples, 92% of TNBC samples exhibited strong positive staining of RARα (p-Ser77), which is significantly higher than that of non-TNBC subtypes (*p* < 0.001) (Fig. 1b), whereas no significant correlations between RARα (p-Ser77) and other clinicopathological variables such as patient age, lymph node status, TNM stage, and metastasis were observed (Supplemental Table 1). These results demonstrated the clinical significance of hyper-phosphorylated RARαS77 serving as a potential molecular target for TNBC patients.

Next, we sought to determine whether RARαS77 is also phosphorylated in TNBC cell lines. RARα (p-Ser77) expression level was detected in three TNBC cell lines (MDA-MB-231, MDA-MB-436, and MDA-MB-453), MCF-7 (PR+/ER+/HER2-), T47D (PR+/ER+/HER2-), and SKBR3 (HER2+) cells. As expected, all three TNBC cell lines exhibited phosphorylated RARαS77, which remained unchanged after the treatment of a selective RARα agonist AM580 [28, 29], whereas the addition of AM580 reduced RARαS77 phosphorylation in RA-sensitive non-TNBC cell lines (Fig. 1c) [10]. This is in accordance with the previous finding that RARα agonists reduced RARα phosphorylation at Ser77 residue in RA-sensitive cells [21]. To determine whether the difference of RARα phosphorylation levels between TNBC and non-TNBC cells is related to their sensitivity to RA, we analyzed TNBC cell proliferation after the treatment of different RARs agonists (pan-RARs agonist ATRA, RARα/β agonist AM80, and RARα agonist AM580). Unlike RA-sensitive cells [10], TNBC cells showed resistance to RARs agonists, which is accompanied by the constant phosphorylation of RARαS77 (Fig. 1d-f, Supplemental Fig. 1). Taken together, these results indicate that the phosphorylation of RARαS77 is associated with RA-resistance of TNBC cells.

### Phosphorylation-defective RARα-mediated suppression of TNBC cell growth in vitro is independent of ligand-activation

**Fig. 2:**
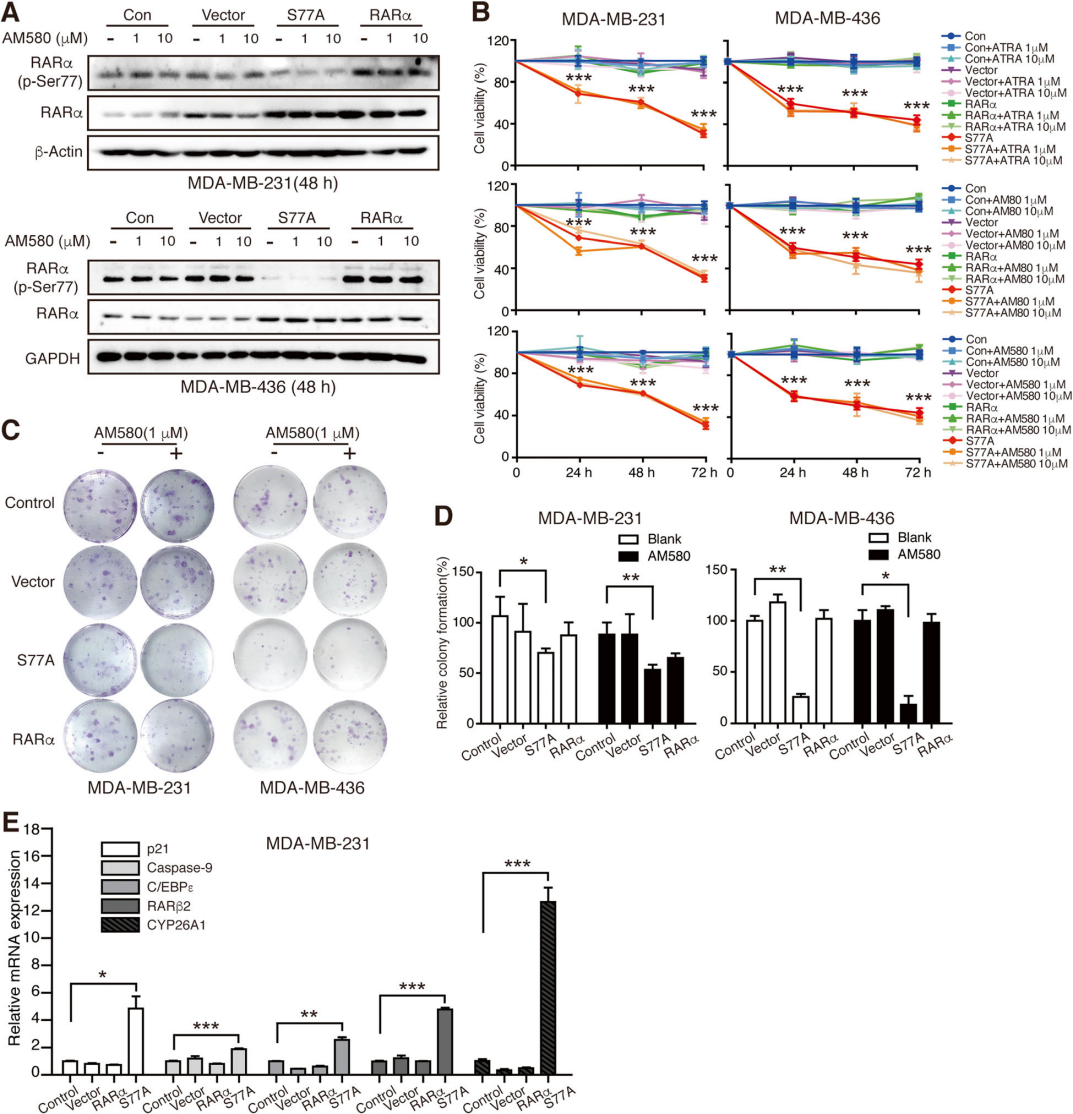
Phosphorylation-defective RARα-mediated suppression of TNBC cell growth in vitro is independent of ligand-activation. **a** Western blotting analysis of RARα (p-Ser77) and total RARα expression levels after overexpression with RARα hypo-phosphorylated mutant (RARαS77A), wild-type RARα, and empty vector. **b** Cell viability after overexpression of RARαS77A, RARα, and empty vector in the presence or absence of different RARα agonists were determined by MTT analysis. RARαS77A versus Control, *** *p* < 0.001. **c** TNBC cells overexpressing RARαS77A, RARα, and empty vector were further cultured with AM580 (0, 1 μM) for 14 days. The colony formation was observed by crystal violet staining. **d** The relative number of colonies in panel C was quantified. **e** qRT-PCR analysis of transcriptional expression of direct RA-target genes. * *p* < 0.05, ** *p* < 0.01, *** *p* < 0.001

Previous reports indicated that RARαS77A, a phosphorylation-defective mutant of RARα, could mimic RARα hypo-phosphorylation and inhibit the proliferation of human squamous carcinoma, osteosarcoma, and AML cells [17–21]. Therefore, we wondered whether RARαS77A can reverse the hyper-phosphorylated status of TNBC cells to overcome RA-resistance and induce proliferation inhibition. After transducing lentiviral RARαS77A, wild-type RARα, and empty vector in TNBC cell lines (Supplemental Fig. 2), western blotting (WB) analysis confirmed the hypo-phosphorylation of RARαS77 and overexpression of RARα receptor (Fig. 2a, Supplemental Fig. 3A). MTT analysis showed that RARαS77A caused a significant decrease of TNBC cell survival regardless of the addition of RARα agonists, whereas overexpression of wild-type RARα in the presence of RARα agonists failed to induce proliferation inhibition (Fig. 2b, Supplemental Fig. 3B). Colony formation assays further proved that RARαS77A reduced TNBC cell growth, independently of RARα agonists (Fig. 2c-d, Supplemental Fig. 3C). Interestingly, the overexpression of RARαS77A alone could activate transcription of direct RA-target genes such as *p21* [30], *Caspase-9* [31], *C/EBPε* [32], *RAR*β_2_ [33], and *CYP26A1* [34] (Fig. 2e), thus suggesting that RARαS77A may override RA-RARα signaling blockade in TNBC cells. The above findings indicate that the decrease of RARαS77 phosphorylation, but not the level of RARα, is crucial for RARα activation and suppression of TNBC growth in vitro.

### RARαS77A induces TNBC cell cycle arrest and apoptosis

**Fig. 3:**
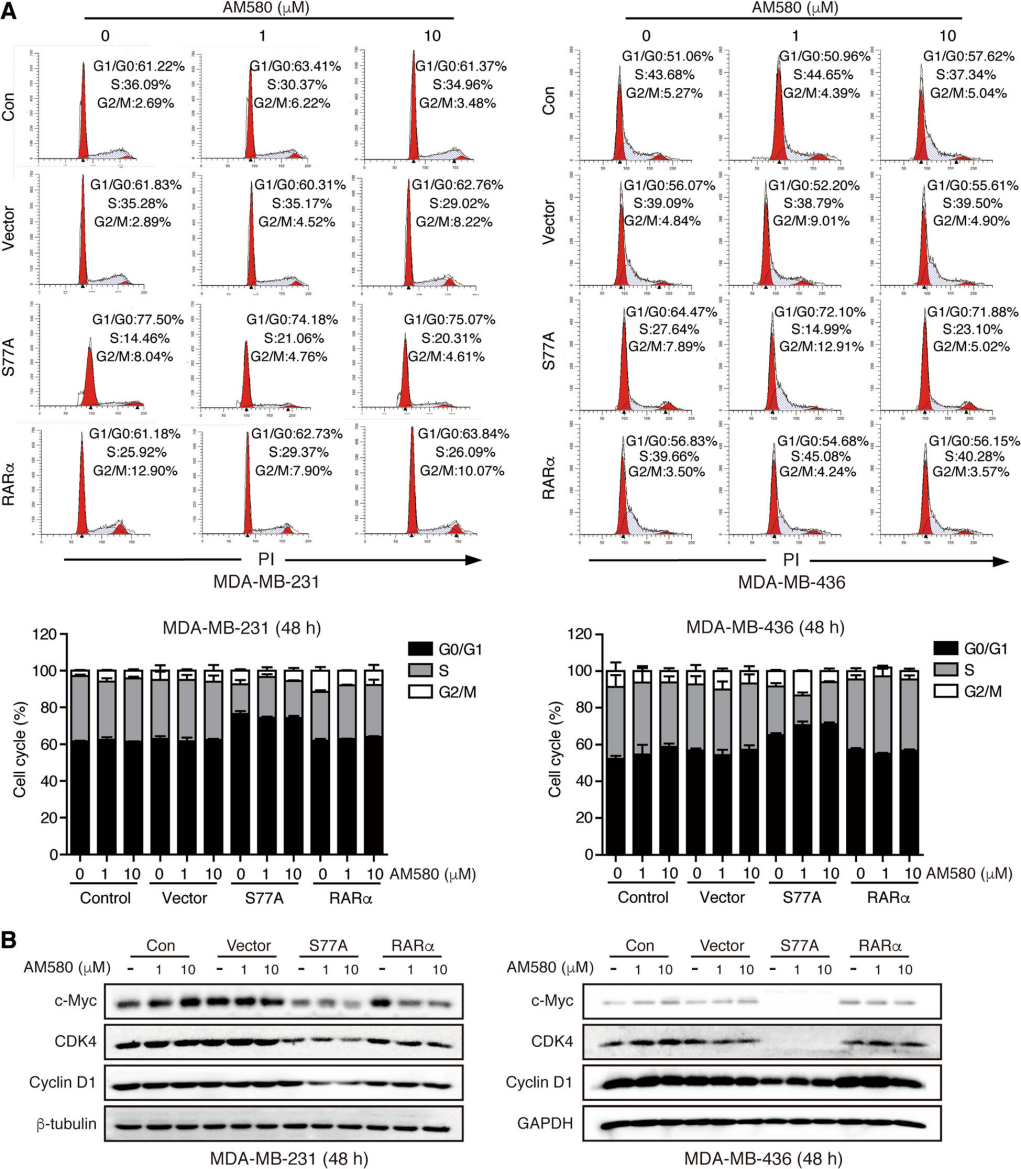
RARαS77A induces TNBC cell cycle arrest. **a** Cell cycle analysis of TNBC cells stably overexpressing RARαS77A, RARα, or vector with or without AM580 treatment. **b** The expression levels of cell-cycle related proteins in TNBC cells overexpressing RARαS77A, RARα, or vector

To explore the mechanism by which RARαS77A inhibited proliferation of TNBC cells, cell cycle analysis was performed to test whether cells overexpressing RARαS77A were arrested in a specific phase. Flow cytometric analysis showed that RARαS77A induced G0/G1 arrest and a concomitant decrease of cell number at the S phase, independently of RARα agonist (Fig. 3a, Supplemental Fig. 4A). This is in line with the previous report that ATRA coordinates G1 arrest by inducing RARα hypo-phosphorylation in APL cells [35]. Consistent with the proliferation data, RARα in the presence of agonist did not cause significant changes in the cell cycle. WB analysis of several cell cycle-related proteins showed that RARαS77A markedly decreased the expression of Cyclin D1, CDK4, and c-Myc (Fig. 3b, Supplemental Fig. 4B). Thus, the phosphorylation status of RARαS77 seems to regulate G1/S phase transition, while hypo-phosphorylated RARαS77A represses cell cycle progression.

**Fig. 4:**
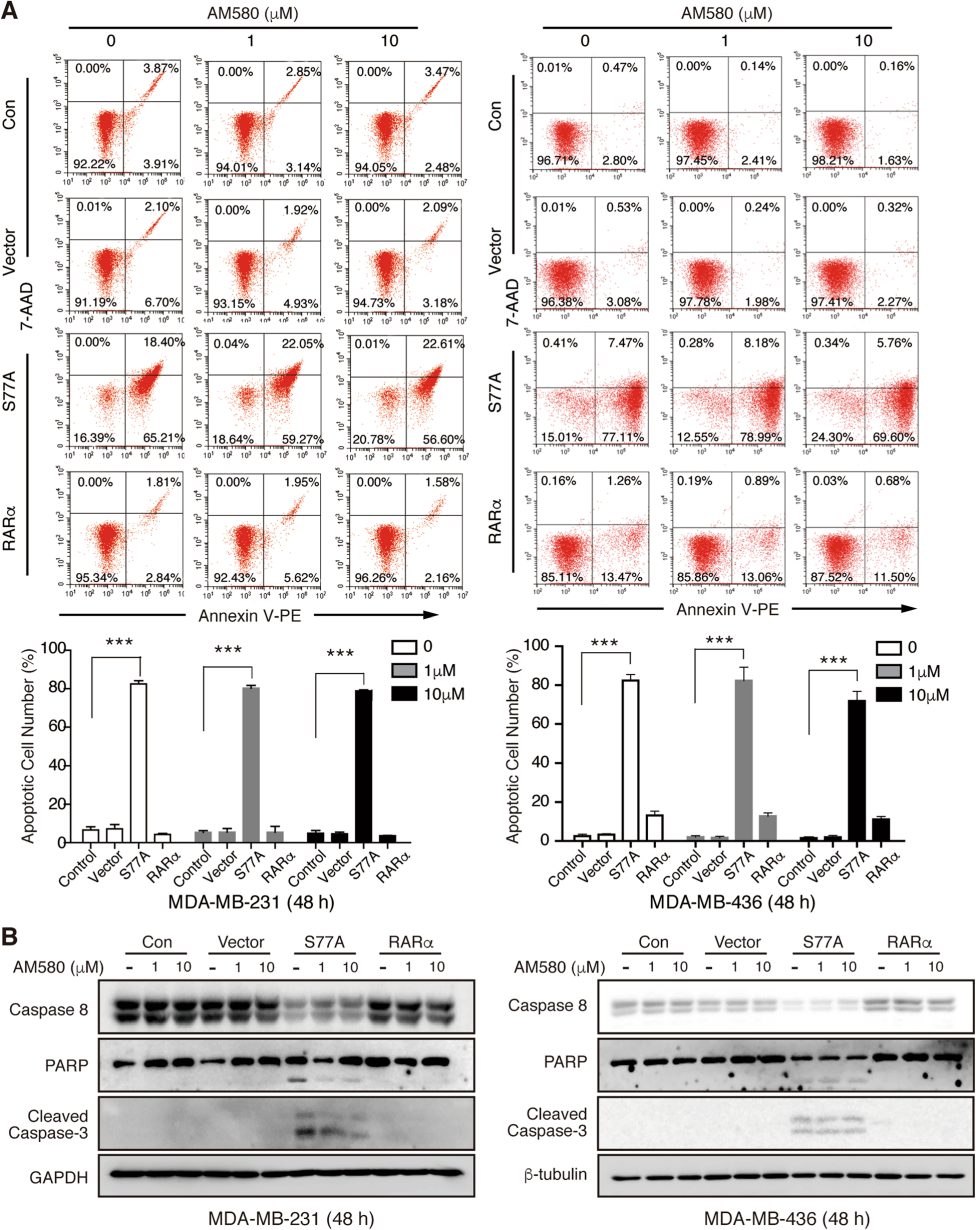
RARαS77A induces TNBC cell apoptosis. **a** Annexin V-APC/7-AAD double-staining assay by flow cytometry was used to detect apoptosis of TNBC cells stably overexpressing RARαS77A, RARα, or vector with or without AM580 treatment. **b** The protein levels of apoptosis-related proteins in TNBC cells overexpressing RARαS77A, RARα, or vector. *** *p* < 0.001

Further to determine whether RARαS77A-induced proliferation inhibition may be related to apoptosis, flow cytometry analysis was performed and results showed that the expression of RARαS77A promoted apoptosis in TNBC cells, regardless of the presence or absence of RARα agonist (Fig. 4a, Supplemental Fig. 4C). WB analysis also confirmed the cleavage and activation of caspase 3/8 and PARP (Fig. 4b, Supplemental Fig. 4d). Collectively, these data suggested that RARαS77A induces cell cycle arrest and apoptosis to inhibit TNBC cell proliferation in vitro.

### RARαS77A activates cytotoxic autophagy in TNBC cells

**Fig. 5:**
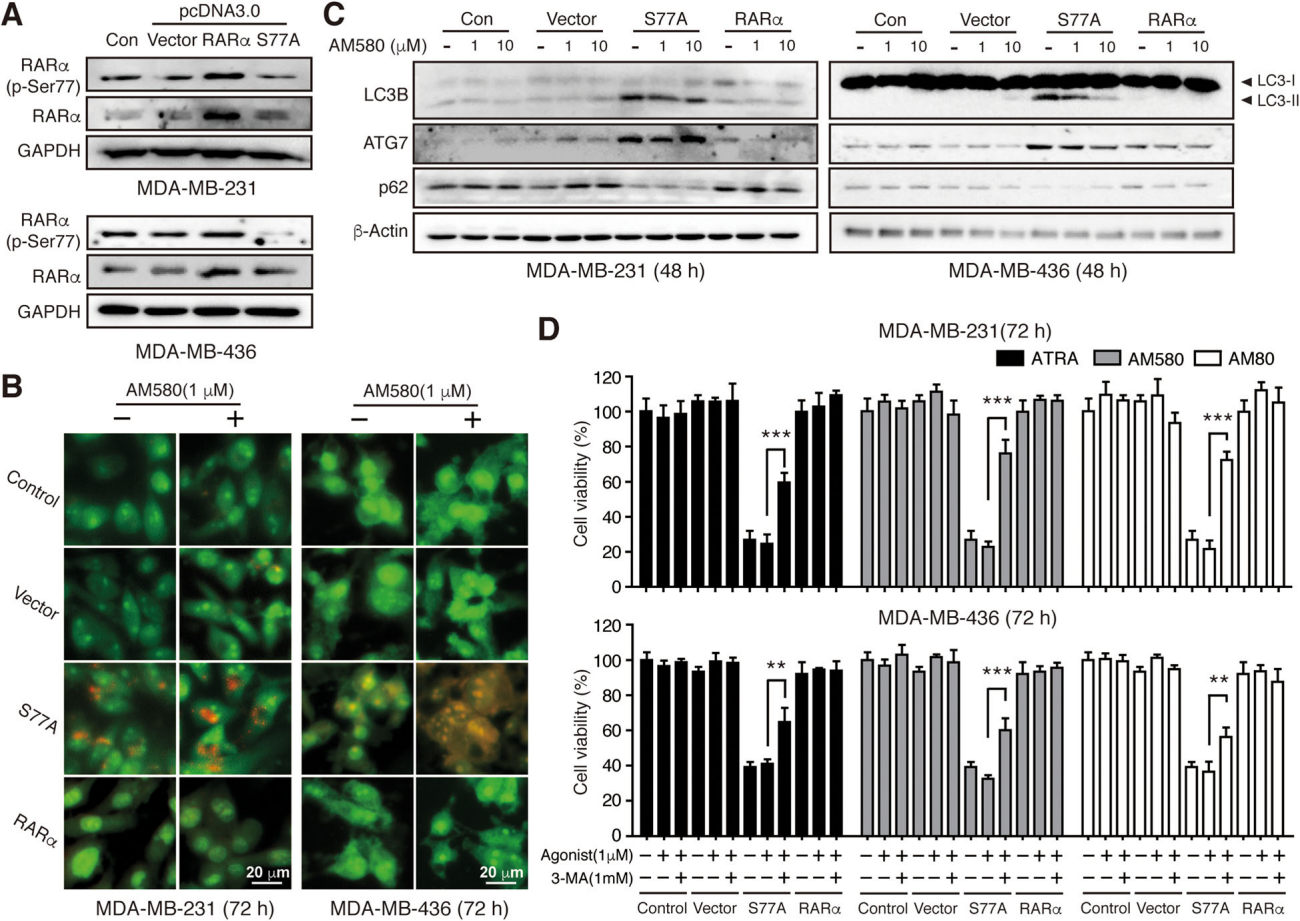
RARαS77A activates cytotoxic-autophagy in TNBC cells. **a** Western blotting analysis of protein expression after transient transfection of pcDNA3.0-vector/RARα/RARαS77A plasmids. **b** AO staining of transfected TNBC cells with or without AM580 for 72 h. Scale bar, 20 μm. **c** Western blotting was performed to detect the changes of autophagy-related proteins. **d** After pretreatment with autophagy inhibitor 3-MA (1 mM) for 2 h, cell viability was assessed in TNBC cells stably overexpressing RARαS77A, RARα, or vector by MTT analysis. ** *p* < 0.01, *** *p* < 0.001

Since activation of RARα induced autophagic flux in RA-sensitive but not in RA-resistant breast cancer cells [36], we asked whether RARαS77A could bypass RARα activation to induce autophagy in TNBC cells as well. To avoid GFP interference from the lentiviral backbone, we transiently transfected pcDNA3.0-vector/RARα/RARαS77A plasmids into all three TNBC cells (Fig. 5a), and observed an increase in the formation of acidic vesicular organelles, accompanied by elevated LC3B-II, ATG7, and decrease of p62 (Fig. 5b-c, Supplemental Fig. 5A-C). As autophagy response can be either cyto-protective or cytotoxic, we next examined the outcome of RARαS77A-induced autophagy. Pre-treatment of autophagy inhibitor 3-MA partially abolished the growth inhibitory effect of RARαS77A (Fig. 5d, Supplemental Fig. 5D), thus suggesting that RARαS77A-induced autophagy is cytotoxic, which may have contributed to the anti-tumor action of RARαS77A.

### RARαS77A suppresses TNBC cell growth in vivo

**Fig. 6:**
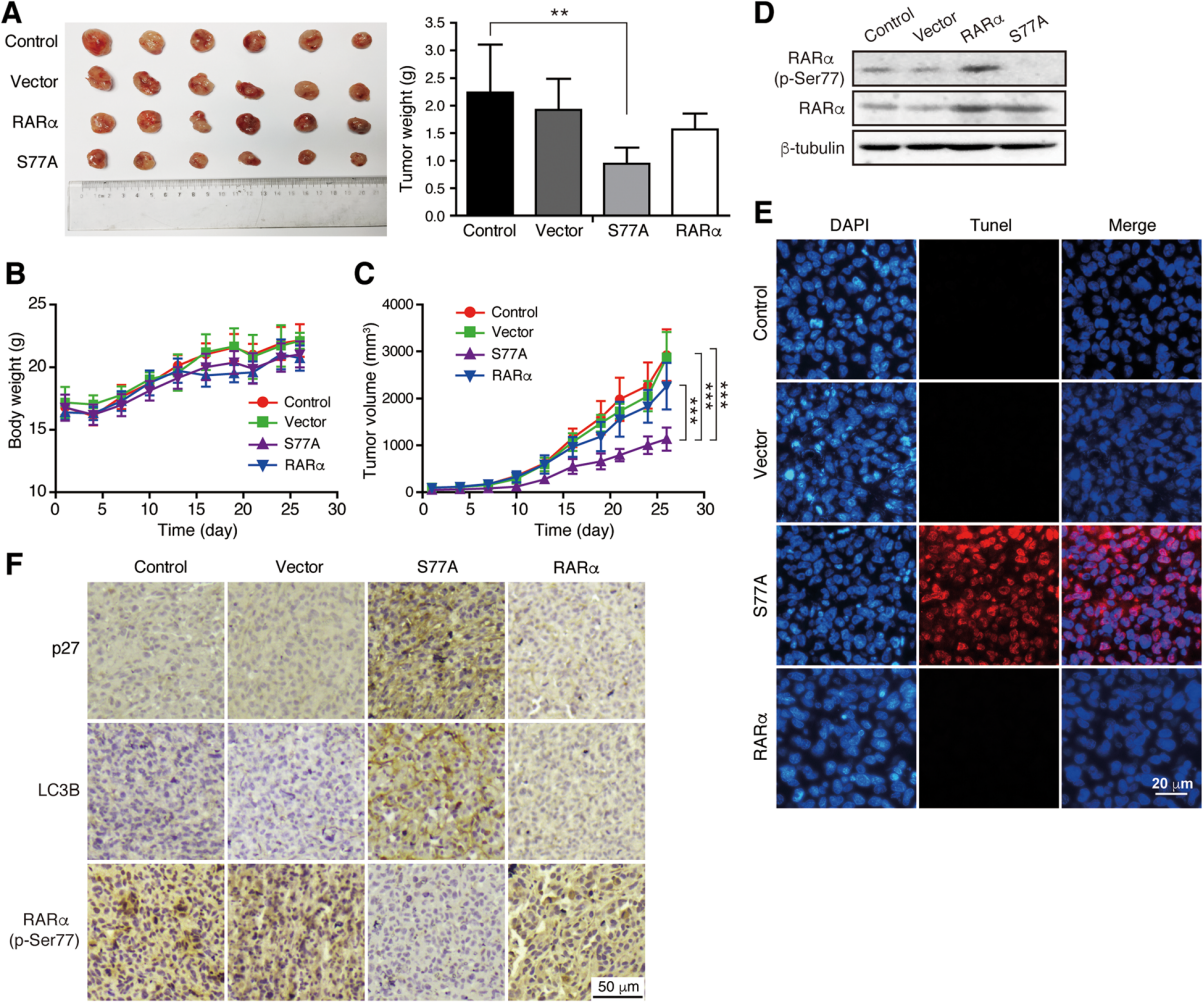
RARαS77A suppresses TNBC cell growth in vivo. **a**-**c** MDA-MB-231 cells stably transfected with RARαS77A, RARα or vector were injected subcutaneously into BALB/c nu/nu mice. The tumor volume and body weight were measured every 3 days. After 26 days, the mice were sacrificed and the tumors were weighed. **d** Tumor tissue homogenate was taken for western blotting to detect RARα (p-Ser77) and RARα protein expression. **e**-**f** TUNEL staining and IHC analysis of tumor xenografts

To further evaluate the roles of RARαS77A on tumor progression in vivo, we performed animal experiments using a nude mouse tumor xenograft model. As shown in Fig. 6a-c, tumors derived from the RARαS77A cell group grew slower and resulted in a smaller size and a lighter weight than those from the control group. Consistent with the in vitro results of this study, hypo-phosphorylation of RARαS77 (Fig. 6d) induced noticeable apoptosis, along with the upregulation of p27 and LC3B expression in tumor tissue sections, as exhibited by TUNEL staining and IHC analysis (Fig. 6e-f). These results suggest that RARαS77A could suppress the growth of TNBC cells in vivo via induction of apoptosis, cell cycle arrest, and autophagy.

### miR-3074-5p mediates the tumor-suppressive function of RARαS77A by targeting DHRS3

**Fig. 7:**
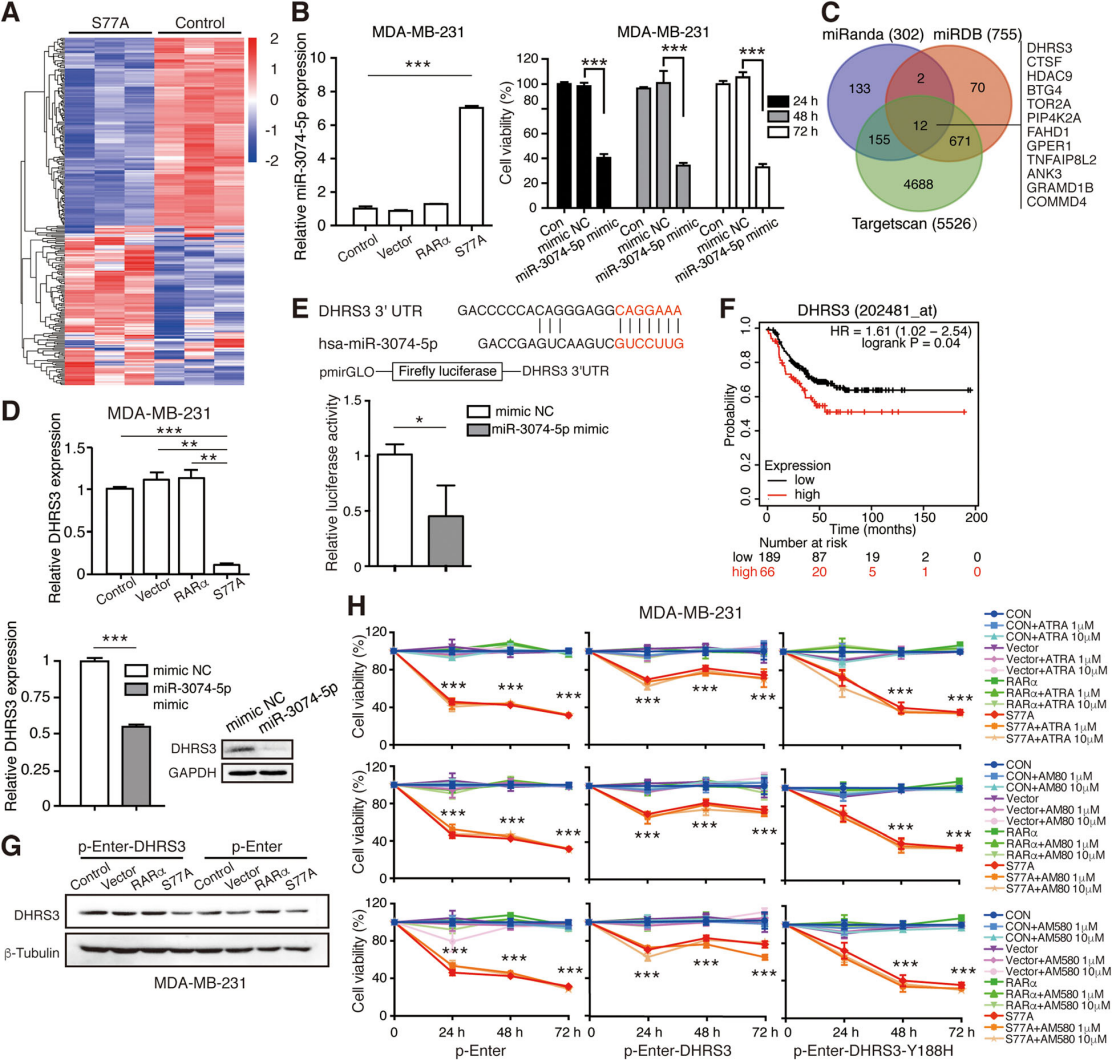
miR-3074-5p mediates the tumor-suppressive function of RARαS77A by targeting DHRS3. **a** Comparison of miRNA expression in RARαS77A-overexpressed and control MDA-MB-231 cells by miRNA sequencing. Each cell was tested in triplicate. **b** qRT-PCR analysis of miR-3074-5p expression in RARαS77A-overexpressed MDA-MB-231 cells, and MTT analysis of cell proliferation after transfections of control mimic and miR-3074-5p mimic. **c** Venn diagram displaying miR-3074-5p computationally predicted targets by three different prediction algorithms: miRanda, TargetScan, and miRDB. **d** qRT-PCR and western blotting analysis of transcriptional and translational levels of DHRS3 after overexpression of either RARαS77A or miR-3074-5p. **e** Schematic representation of DHRS3 3’UTR demonstrating putative miRNA target site. Relative luciferase activity of co-transfection of miR-3074-5p with the WT 3′-UTR of DHRS3 in 293FT cells. **f** Kaplan-Meier representations of the probabilities of recurrence-free survival according to the expression levels of DHRS3 in TNBC patients. A log-rank test was used to evaluate significance. **g** Cells were transiently transfected with p-Enter vector or p-Enter-DHRS3, and the DHRS3 protein level was detected by western blotting. **h** MTT analysis of cell viability of MDA-MB-231 cells overexpressing wild-type DHRS3 or DHRS3-Y188H mutant, in the presence or absence of different RARs agonists. * *p* < 0.05, ** *p* < 0.01, *** *p* < 0.001

Given that RARα is a transcription factor that participates in regulating transcription of various genes as well as miRNAs [9, 25], we were thus inspired to investigate whether the anti-tumor efficacy of RARαS77A is attributed to activating transcription of functional miRNAs. We performed a miRNA sequencing analysis of MDA-MB-231 cells overexpressing RARαS77A. Results identified 126 up-regulated miRNAs and 144 down-regulated miRNAs, compared to control cells (Fig. 7a, Supplemental Data). Among these miRNAs, miR-3074-5p, a regulator of oncogenic cAMP-responsive element binding-protein [37], was highly expressed and validated by qRT-PCR (Fig. 7b). To explore the biological function of miR-3074-5p in TNBC, we overexpressed miR-3074-5p mimic or mimic-NC in MDA-MB-231 cells. MTT analysis showed that miR-3074-5p impaired MDA-MB-231 cell growth by decreasing cell viability (Fig. 7b), thus implying a potential tumor-suppressive role of miR-3074-5p.

To explore which mRNA target of miR-3074-5p mediates the antitumor efficacy of RARαS77A, candidate genes retaining miR-3074-5p-binding sequences were predicted using miRanda, miRDB, and Targetscan (Fig. 7c). Among these predicted targets, the dehydrogenase/reductase member 3 (DHRS3, also known as retSDR1) was the candidate with the most likely predicted binding site and is known to be involved in maintaining the cellular supply of retinol metabolites [38, 39]. qRT-PCR and WB analysis confirmed that both RARαS77A and miR-3074-5p decreased transcription and expression of DHRS3 (Fig. 7d). To further demonstrate that miR-3074-5p directly regulates expression of DHRS3 mRNA through binding to its 3’UTR, wild-type (pmirGLO-DHRS3–3’UTR-wt) reporter vectors were co-transfected with either miR-3074-5p mimics or mimic-NC into 293FT cells. Luciferase activity of the 3’UTR construct of DHRS3 was attenuated by miR-3074-5p overexpression (Fig. 7e), thus suggesting that DHRS3 was a direct target of miR-3074-5p.

By using the online database Kaplan–Meier plotter, we found that DHRS3 is an unfavorable prognosis factor and is negatively associated with TNBC patients’ overall survival (Fig. 7f). Therefore, by overexpressing DHRS3 in cells with hypo-phosphorylated RARαS77 (Fig. 7g), we sought to investigate whether DHRS3 might offset the anti-tumor action of RARαS77A. As expected, DHRS3 partially attenuated the proliferation-inhibitory effect of RARαS77A (Fig. 7h, Supplemental Fig. 6). This pro-survival function of DHRS3 is presumably related to its reductase activity, as the overexpression of DHRS3 with a mutated catalytic residue Tyr188 (DHRS3-Y188H) [39] failed to abolish RARαS77A’s action (Fig. 7h). Taken together, these findings indicate that miR-3074-5p mediated (at least in part) the tumor-suppressive effect of RARαS77A by targeting DHRS3 in TNBC.

### CDK7 inhibitor THZ1 suppresses TNBC growth via RARαS77-DHRS3 signaling

**Fig. 8:**
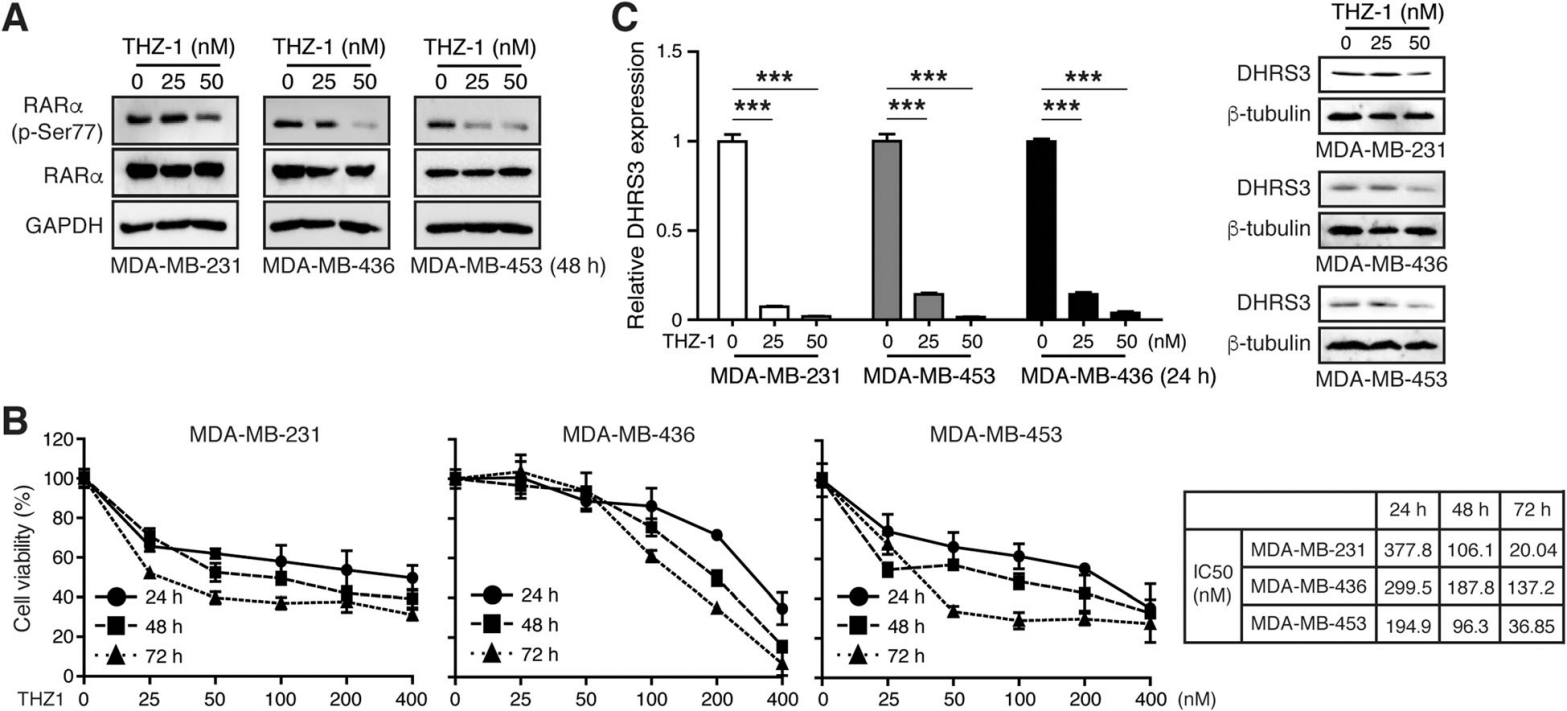
CDK7 inhibitor THZ1 suppresses TNBC growth via RARαS77-DHRS3 signaling. **a** Western blotting analysis of RARα (p-Ser77) and RARα protein expression levels after treatment of THZ1 for 48 h. **b** TNBC cell viability was assessed after THZ1 treatment by MTT analysis and IC_50_ values were calculated. **c** qRT-PCR and western blotting analysis of DHRS3 levels after THZ1 treatment

Because CDK7 is the major subunit of CAK complex responsible for phosphorylation of RARαS77 [40], we further addressed whether targeting CDK7 could be a treatment strategy to reduce phosphorylation of RARαS77 in TNBC cells. As expected, a highly specific covalent CDK7 inhibitor THZ1 [41] markedly suppressed RARαS77 phosphorylation as well as TNBC cell growth in vitro (Fig. 8a-b). This anti-tumor action of THZ1 may be attributed to the down-regulation of DHRS3 (Fig. 8c). Thus, targeting the phosphokinase of RARαS77 may be a feasible approach to treat RA-resistant TNBC.

## Discussion

Study showed that a high proportion of Luminal/ER+ carcinomas are RA sensitive, while triple-negative (basal) tumors tend to be retinoid resistant [10]. One possible reason for this RA-resistance is the subcellular distribution of RA. The delivery of RA to RARs in the nucleus by cellular retinoic acid binding protein 2 (CRABP2) leads to inhibition of cell proliferation, apoptosis, invasion, and metastasis, whereas delivery of RA to peroxisome proliferator activated receptor beta (PPARβ) by FABP5 increases cell proliferation and causes RA resistance [42–44]. Higher expression of FABP5 is found in ER/PR-negative breast cancers, which competes with CRABP2 for RA ligand binding, and correlates with high histological grade and a poor prognosis [45]. Therefore, finding ways to activate RARs in a ligand-independent manner may bypass the unwanted pro-survival effect of RA. Here, we showed that RARαS77A, a mutant that mimicked RARα hypo-phosphorylation, could inhibit proliferation of TNBC both in vitro and in vivo. This antitumor effect was mediated by the induction of apoptosis, cell cycle arrest, and cytotoxic autophagy. Interestingly, RARαS77A alone could activate RA-target gene transcription, while the addition of retinoids did not enhance RARαS77A’s efficacy (Figs. 2, 3, 4, 5 and 6). This is possibly due to the conformation change of RARα where hypo-phosphorylated RARα dissociated from transcriptional repressor and associated with coactivator [21]. Therefore, RARαS77A functioned as an activated form of RARα independent of RA stimulation, thus evading the pro-survival impact of RA on TNBC.

In this study, we explored the biological function of miR-3074-5p and its interaction with the RARα signaling pathway in TNBC. Previously, miR-3074-5p was shown to be associated with favorable prognosis of papillary renal cell carcinoma patients [46], and acted primarily to inhibit cell proliferation and neuronal differentiation of oligodendrocyte precursors [47]. We found that RARαS77A-induced transcription of miR-3074-5p inhibited TNBC cell proliferation, at least in part by directly targeting DHRS3 (Fig. 7). However, exactly how RARαS77A regulated miR-3074-5p transcription remains to be determined. As RARα is a known transcription factor that binds to RARE in the promoters of RA-target genes to modulate gene transcription [21], we suspect that RARαS77A may activate miR-3074-5p transcription in a similar fashion. Indeed, in ERα + MCF-7 cells, ligand-dependent activation of RARα increased its binding to the RARE regions of miR-21 promoter and enhanced miR-21 transcription [9]. Further research on the putative RARE binding sites of miR-3074-5p promoter by chromatin immunoprecipitation are warranted. According to the miRNA microarray data, in addition to the transactivation of miR-3074-5p, RARαS77A also up-regulated tumor-suppressive miRNA such as miR-589-5p, while down-regulated oncogenic miRNA such as miR-181a-3p (Supplemental Data). These altered miRNAs might have contributed to the anti-tumor action of RARαS77A as well, thus explaining why miR-3074-5p’s target DHRS3 only partially counteracted RARαS77A-induced growth arrest, and more work is required before we can fully understand the interplay between RARα and these altered miRNAs.

This study also identified the molecular mechanism underlying miR-3074-5p’s function in TNBC by discovering a direct target gene, DHRS3. DHRS3 is an enzyme strongly induced by RA in human neuroblastoma [48] and leukemic monocyte cell lines [49], which mainly catalyzes the reduction of all-trans-retinal, an opposite reaction of RA formation [39, 50]. However, the function of DHRS3 in TNBC and its association with RARα remains poorly understood. We demonstrated here that DHRS3 is negatively correlated with TNBC patients’ overall survival, which is consistent with the previous finding of frequent amplification of DHRS3 in the intermediate/high-risk group of papillary thyroid carcinomas [51], thus suggesting a potential oncogenic function of DHRS3. By transcriptional activation of miR-3074-5p that directly target DHRS3, RARαS77A inhibited TNBC cell proliferation in vitro (Fig. 7). Nevertheless, our data hinted that DHRS3 may possess catalytic functions other than a retinal reductase, as the reduction of DHRS3 leading to possible elevated RA concentration did not enhance the antitumor action of RARαS77A. Since DHRS3 also participated in the metabolism of other endogenous compounds, such as androstenedione, estrone, and DL-glyceraldehyde, and in the biotransformation of xenobiotics [52], it is reasonable to investigate this enzyme from another point of view in addition to its well-established functions, and more research is needed to clarify the exact role of DHRS3 in RARαS77A-mediated anti-TNBC activity.

Our findings may have significant clinical implications for the development of anti-TNBC agents, by either mimicking the structure-conformation of hypo-phosphorylated RARαS77 or directly targeting its phosphokinase to overcome RA resistance, whereas miR-3074-5p and DHRS3 levels may also serve as potential diagnostic and/or prognostic markers and therapeutic targets in TNBC patients.

## Conclusions

In summary, we demonstrated for the first time, that hyper-phosphorylation of RARαS77 is associated with RA-resistance while hypo-phosphorylated RARαS77A functioned in an RA-independent manner to suppress TNBC progression in vitro and in vivo, by transactivating miR-3074-5p and reducing DHRS3. Our study provides new insights into the role and molecular mechanism of RARα signaling in TNBC.

## Supplementary Information

**Additional file 1: Supplemental Table 1.** Association between RAR⍺(p-Ser77) and clinical characteristic of breast cancer. **Supplemental Figure 1.** RARαS77 is constantly phosphorylated in MDA-MB-453 cells, which is associated with RA-resistance. **Supplemental Figure 2.** Lentiviral overexpression of RARαS77A and RARα in TNBC cells. **Supplemental Figure 3.** RARαS77A suppresses MDA-MB-453 cell growth in vitro. **Supplemental Figure 4.** RARαS77A induces cell cycle arrest and apoptosis in MDA-MB-453 cells. **Supplemental Figure 5.** RARαS77A induces cytotoxic-autophagy in MDA-MB-453 cells. **Supplemental Figure 6.** DHRS3 mediates the inhibitory effect of RARαS77A.

**Additional file 2.** Differential miRNAs.

## Data Availability

All data generated or analyzed during this study are included in this published article and its supplementary information files.

## Abbreviations

TNBC: Triple negative breast cancer
RARα: Retinoic acid receptor alpha
CDK7: Cyclin-dependent kinase 7
CAK: Cyclin-dependent kinase-activating kinase
miRNA: microRNA
ER: Estrogen receptor
PR: Progesterone receptor
PI: Propidium iodide
SD: Standard deviation
