# Supplementary material for: Inhibition of retinoic acid receptor α phosphorylation represses the progression of triple-negative breast cancer via transactivating miR-3074-5p to target DHRS3
Source: J Exp Clin Cancer Res. 2021 Apr 26;40:141. doi: 10.1186/s13046-021-01941-7 (PMC8074416; doi:10.1186/s13046-021-01941-7)
Supplement: Supplementary file 1 — Additional file 1: Supplemental Table 1. Association between RAR⍺(p-Ser77) and clinical characteristic of breast cancer. Supplemental Figure 1. RARαS77 is constantly phosphorylated in MDA-MB-453 cells, which is associated with RA-resistance. Supplemental Figure 2. Lentiviral overexpression of RARαS77A and RARα in TNBC cells. Supplemental Figure 3. RARαS77A suppresses MDA-MB-453 cell growth in vitro. Supplemental Figure 4. RARαS77A induces cell cycle arrest and apoptosis in MDA-MB-453 cells. Supplemental Figure 5. RARαS77A induces cytotoxic-autophagy in MDA-MB-453 cells. Supplemental Figure 6. DHRS3 mediates the inhibitory effect of RARαS77A. [file 13046_2021_1941_MOESM1_ESM.docx]

**Supplemental Information**

**SUPPLEMENTAL FIGURES AND LEGENDS**


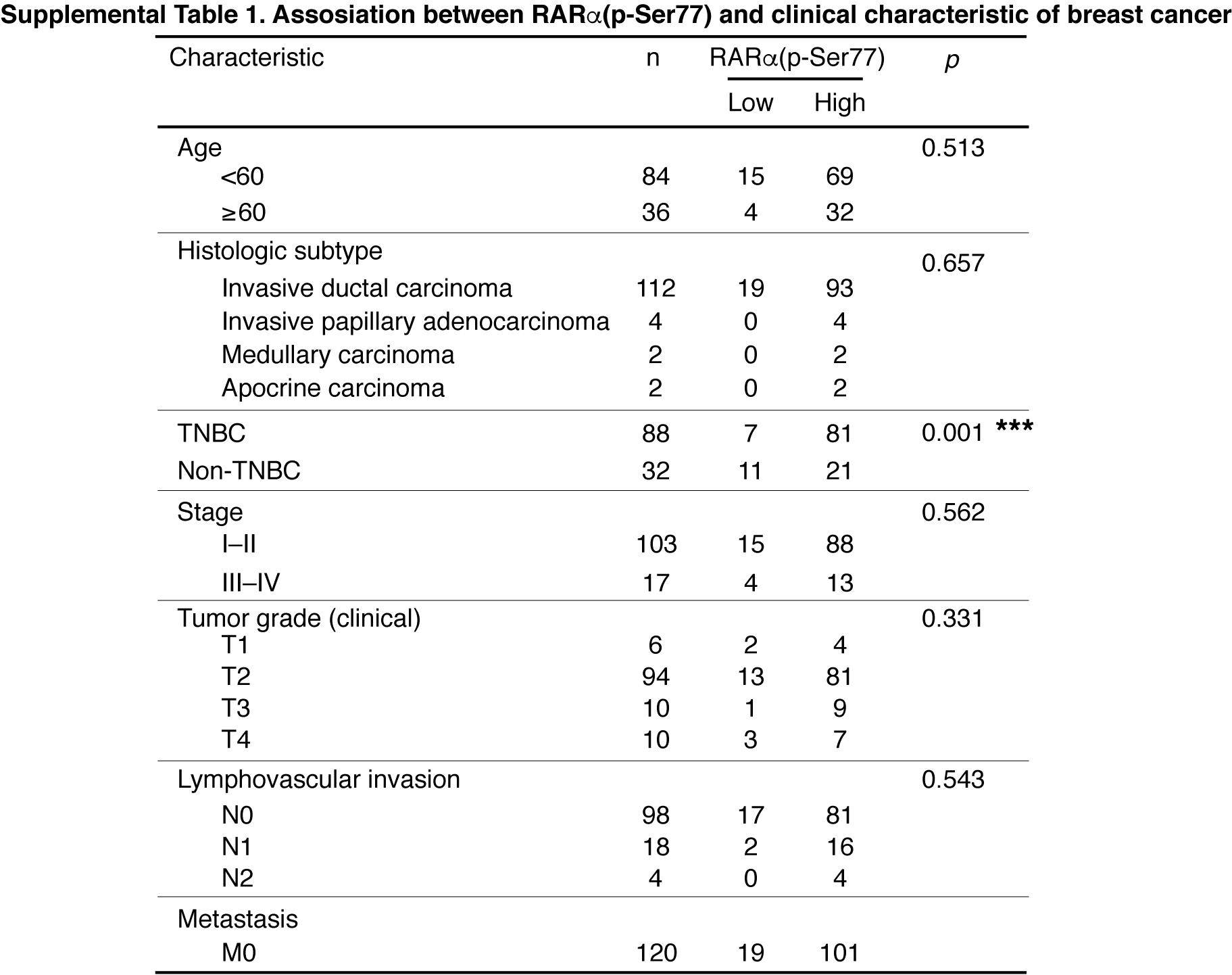


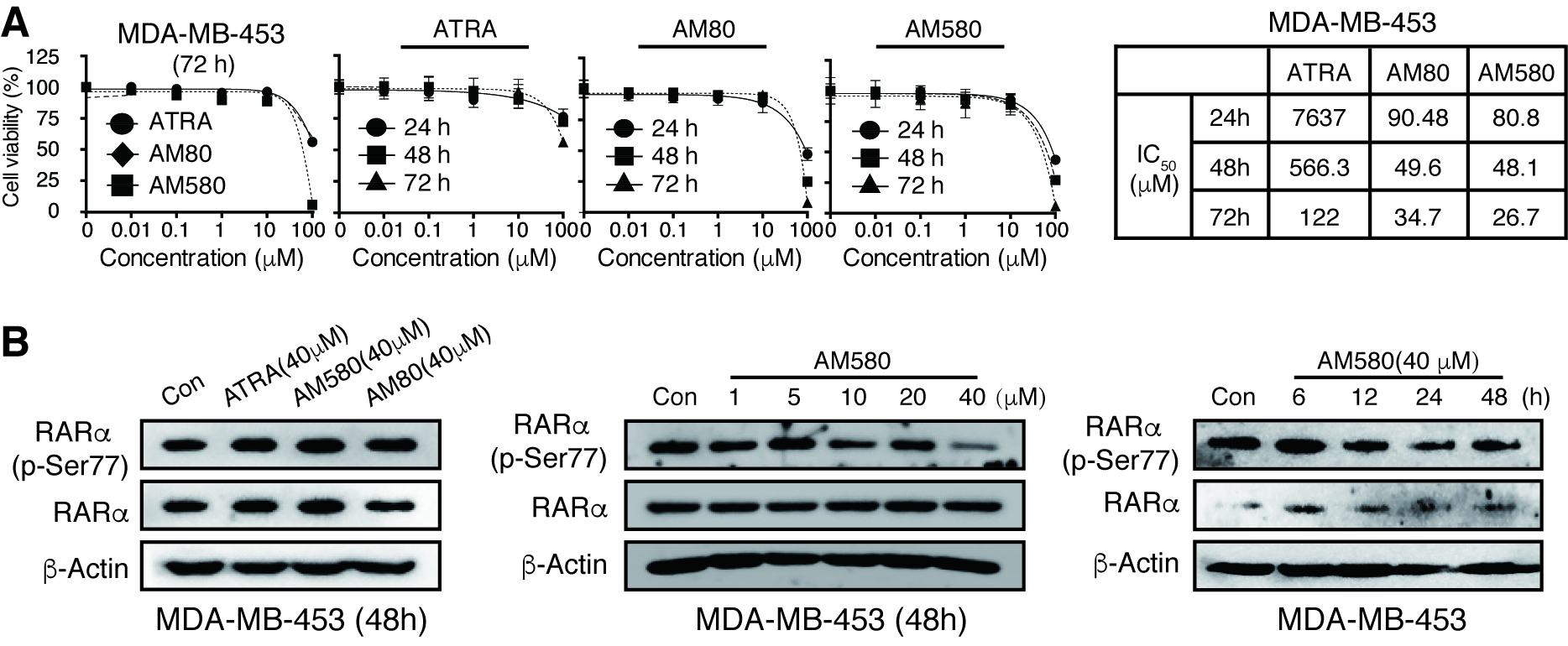


**Supplemental Figure 1.** RARαS77 is constantly phosphorylated in MDA-MB-453 cells, which is associated with RA-resistance. (A) MDA-MB-453 cells were treated with pan-RARs agonist ATRA, RARα/β agonist AM80, and RARα agonist AM580 for indicated concentrations and time. Cell viability was assessed by MTT analysis and IC_50_ values were calculated. (B) After treatment of different RARs agonists for indicated concentrations and time, RARα (p-Ser77) and total RARα expression levels were detected by western blotting.


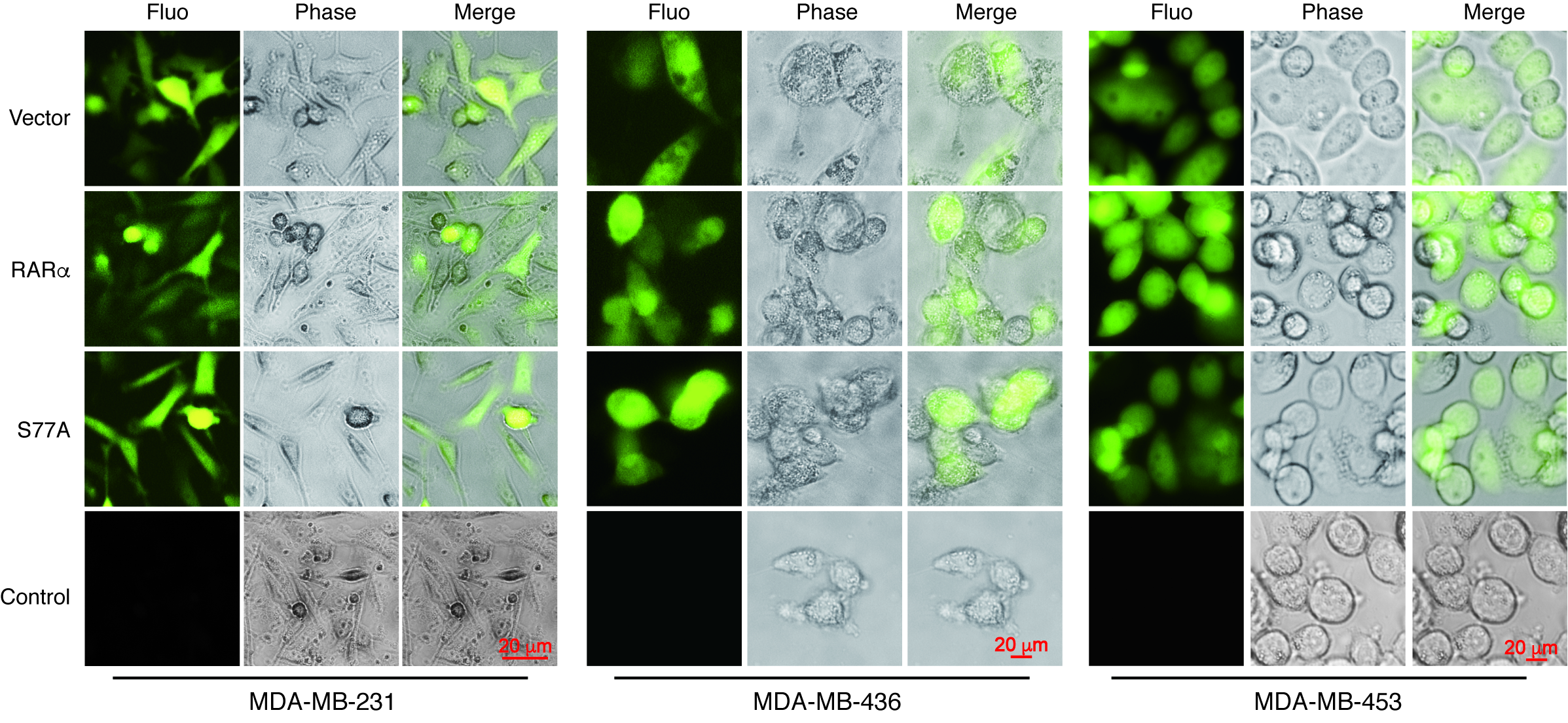


**Supplemental Figure 2.** Lentiviral overexpression of RARαS77A and RARα in TNBC cells. Expression levels of lentiviral RARαS77A-GFP, RARα-GFP and vector in TNBC cells were visualized under low-resolution fluorescence microscope. The expression levels were at least ≥ 70%. Scale bar, 20 μm.


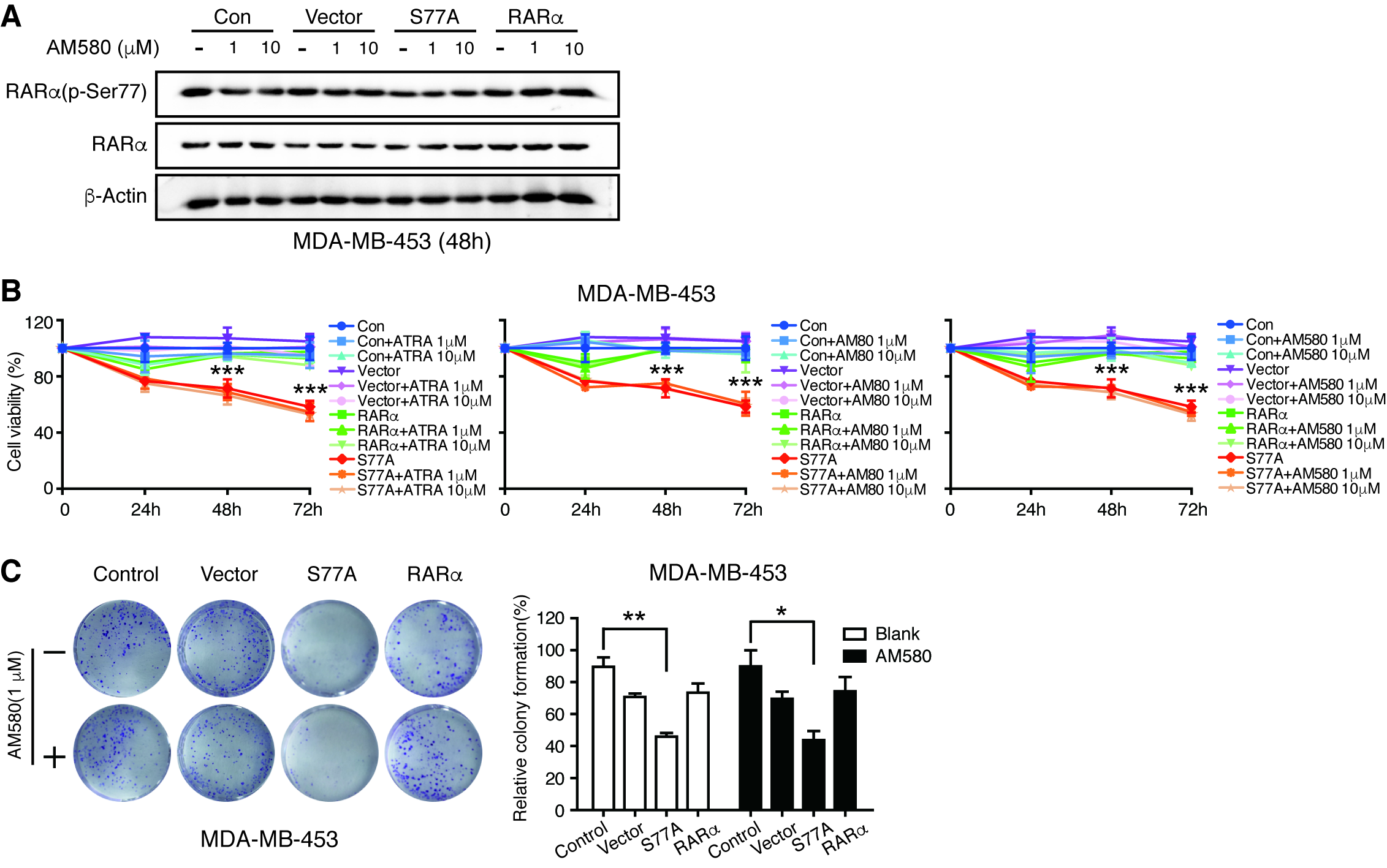


**Supplemental Figure 3.** RARαS77A suppresses MDA-MB-453 cell growth *in vitro*. (A) Western blotting analysis of RARα (p-Ser77) and total RARα expression levels after overexpression with RARαS77A, wild-type RARα, or empty vector. (B) MDA-MB-453 cell viability after overexpression of RARαS77A, RARα, or empty vector in the presence or absence of different RARα agonists were determined by MTT analysis. RARαS77A versus Control, *** *p* < 0.001. (C) MDA-MB-453 cells overexpressing RARαS77A, RARα, or empty vector were further cultured with AM580 (0, 1 μM) for 14 days. The colony formation was observed by crystal violet staining. * *p* < 0.05, ** *p* < 0.01.


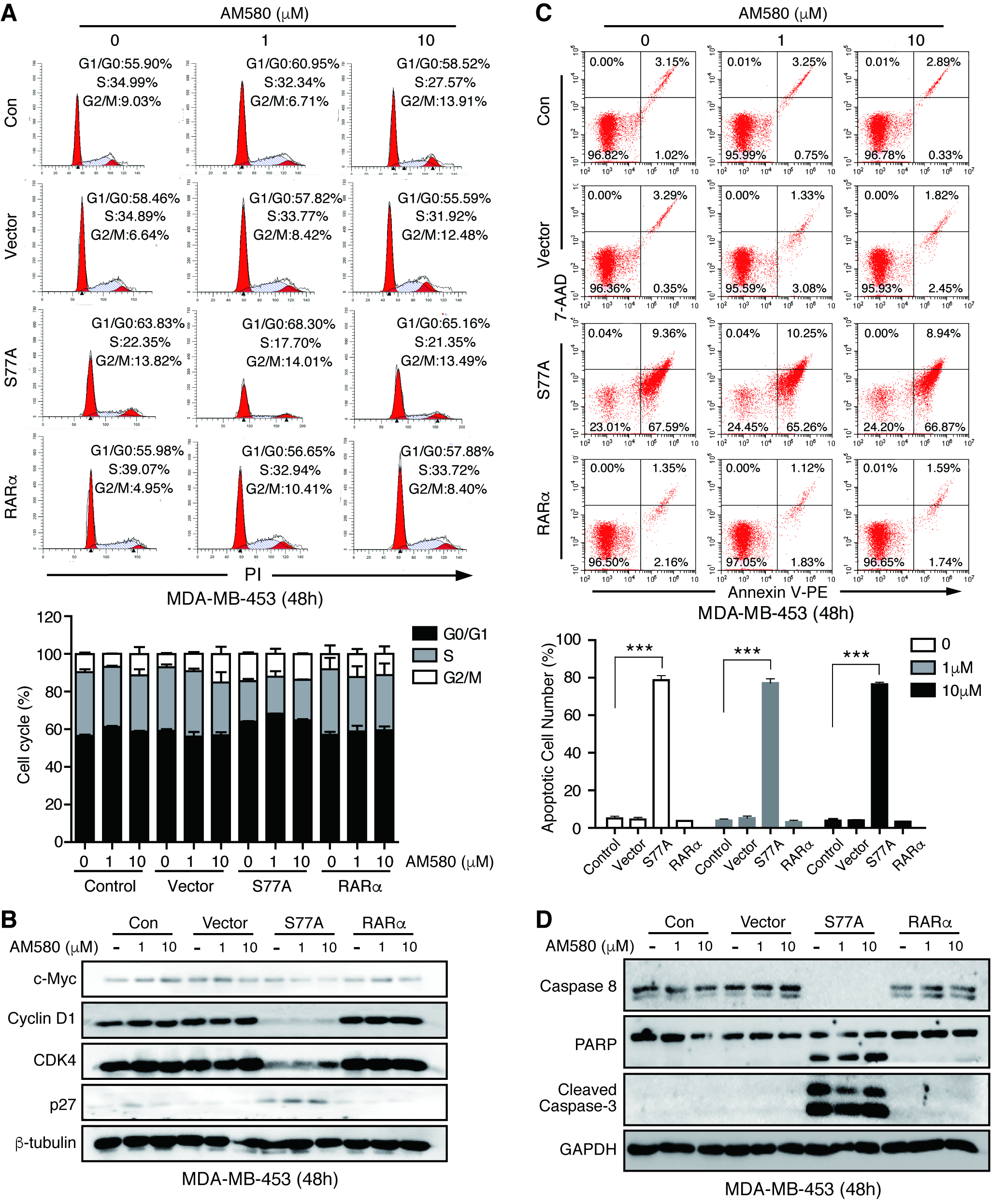


**Supplemental Figure 4.** RARαS77A induces cell cycle arrest and apoptosis in MDA-MB-453 cells. (A) Cell cycle analysis of MDA-MB-453 cells stably overexpressing RARαS77A, RARα, or vector with or without AM580 treatment. (B) The expression levels of cell-cycle related proteins in MDA-MB-453 cells overexpressing RARαS77A, RARα, or vector. (C) Annexin V-APC/7-AAD double-staining assay by flow cytometry were used to detect apoptosis of MDA-MB-453 cells stably overexpressing RARαS77A, RARα, or vector with or without AM580 treatment. (D) The expression levels of apoptosis-related proteins in MDA-MB-453 cells overexpressing RARαS77A, RARα, or vector. *** *p* < 0.001.


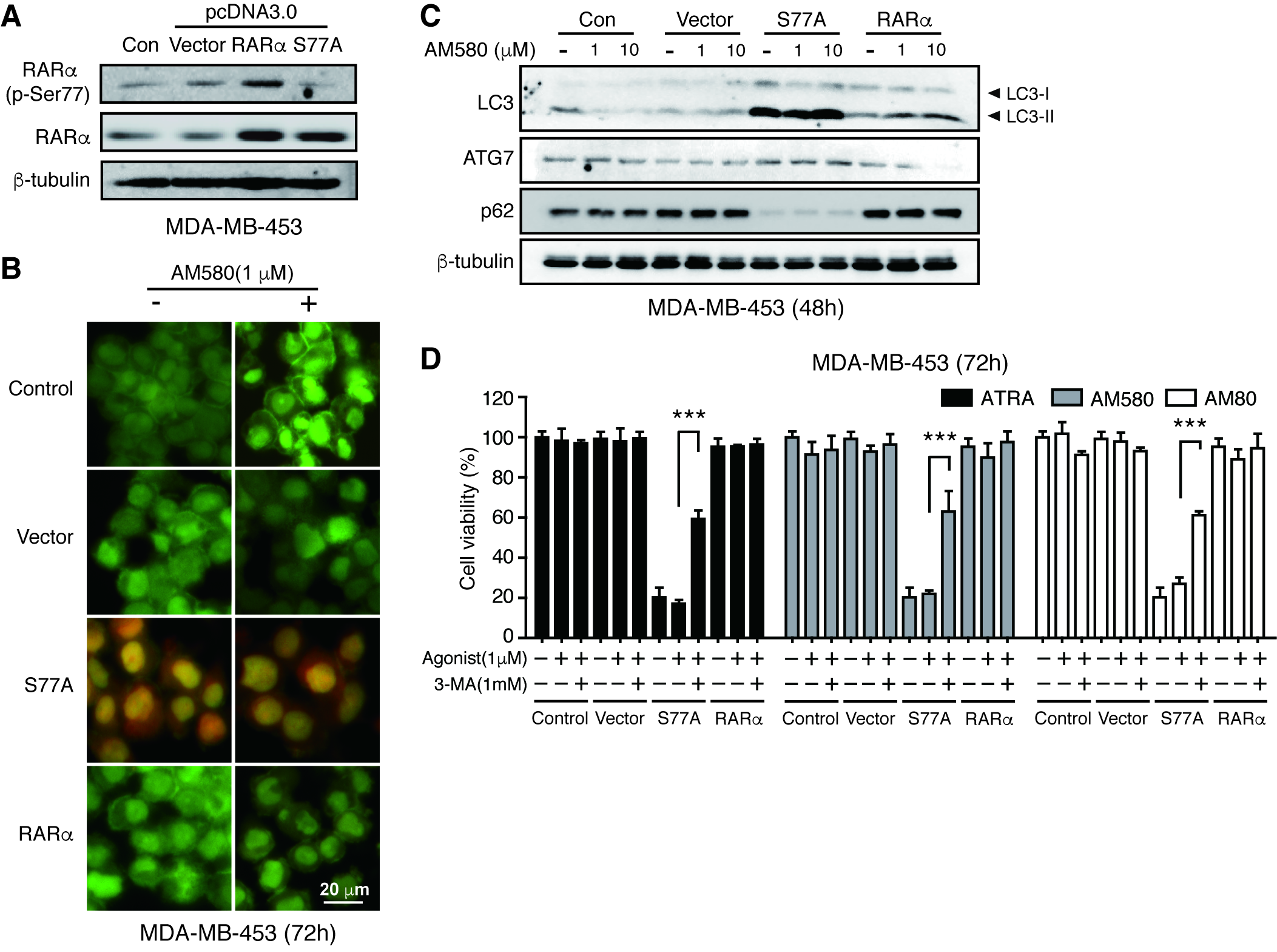


**Supplemental Figure 5.** RARαS77A induces cytotoxic-autophagy in MDA-MB-453 cells. (A) Western blotting analysis of protein expression after transient transfection of pcDNA3.0-vector/RARα/RARαS77A. (B) AO staining of transfected MDA-MB-453 cells with or without AM580 for 72 h. Scale bar, 20 μm. (C) Western blotting was performed to detect the changes of autophagy-related proteins. (D) After pretreatment with autophagy inhibitor 3-MA (1 mM) for 2 h, cell viability was assessed in MDA-MB-453 cells stably overexpressing RARαS77A, RARα, or vector by MTT analysis. *** *p* < 0.001.


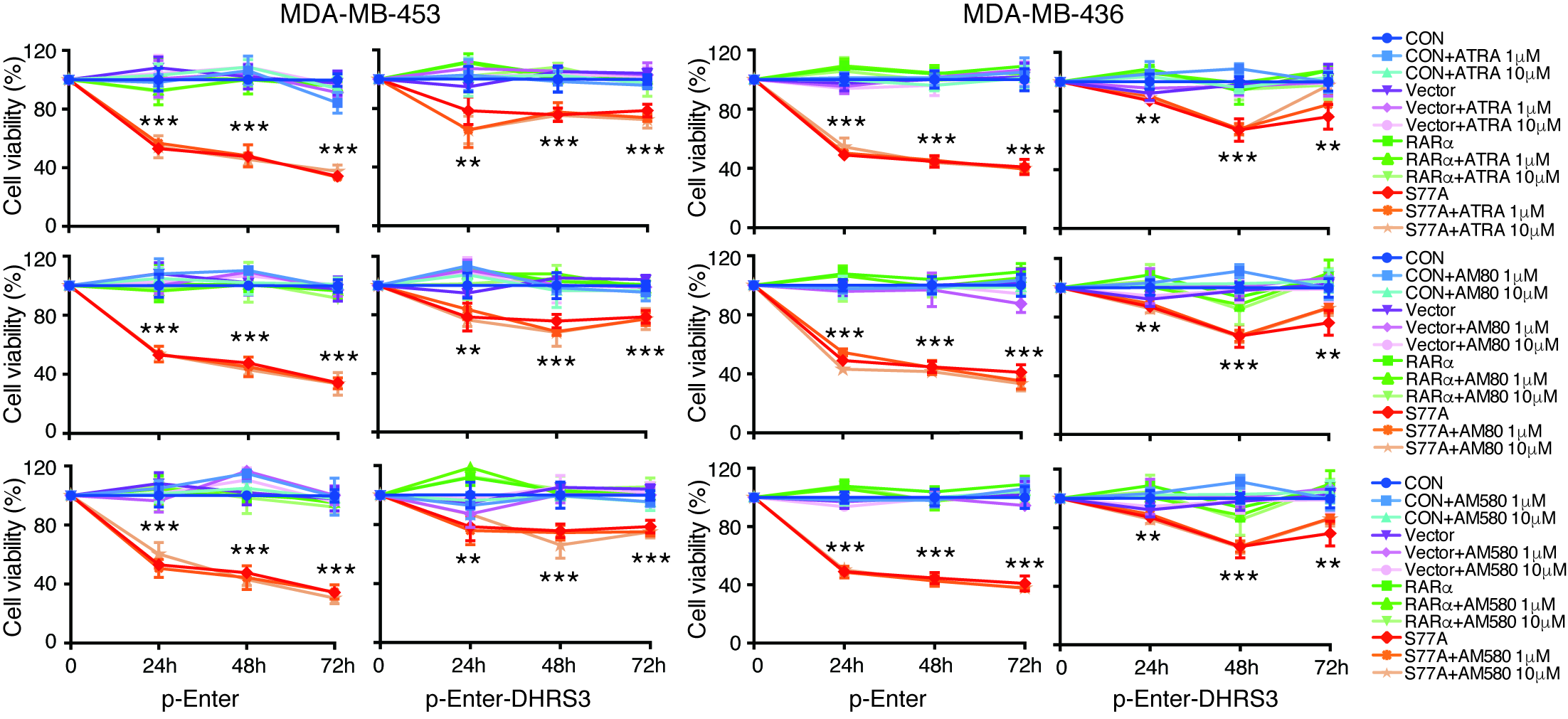


**Supplemental Figure 6.** DHRS3 mediates the inhibitory effect of RARαS77A. Cell viability of MDA-MB-453 and MDA-MB-436 cells overexpressing empty vector or wild-type DHRS3 in the presence or absence of different RARs agonists by MTT analysis. ** *p* < 0.01, *** *p* < 0.001.

**SUPPLEMENTAL METHODS**

**Chemicals and reagents**

Antibody against cyclin D1 (#2261-1) was purchased from Abcam (Cambridge, UK). The antibody against RARα (sc-515796) was from Santa Cruz Biotechnology (CA, USA). The antibody against RARα (p-Ser77) was from Sigma-Aldrich (St. Louis, MO, USA). And antibodies against cleaved caspase-3 (Asp175, #9664), caspase-8 (#4790), PARP (#9532), c-Myc (#9402), CDK4 (#12790), ATG7 (#8558), SQSTM1/p62 (#5114), LC3B (#3868s), β-actin (#4970), β-tubulin (#2128), GAPDH (#5174) were purchased from Cell Signaling Technologies (Danvers, MA, USA). Horseradish peroxidase-conjugated secondary antibodies were purchased from Bio-Rad (Hercules, CA, USA).

**Tissue microarrays**

Tissue microarrays (TMA) slide containing 60 breast cancer patient samples in duplicate (BR1201b) was purchased from Xi’an Avilabio Co. Ltd. TMA were immune-stained with anti-RARα (pSer77) antibody. The scoring method was as follows: 10 random fields of view were selected for each tissue section, and semi-quantitative scoring was performed for tissue staining in each field of view. Positive cell rate integral method: 0, no positive cells or < 10% positive cells; 1, positive cells accounted for 10%~ 25%; 2, positive cells accounted for 25%~ 50%; 3, positive cells accounted for 50%~ 75%; 4, the proportion of positive cells > 75%. Dyeing strength integral method: 0, cells without staining; 1, color is light yellow; 2, color is brown-yellow; 3, color is tan. The total score is the sum of positive cell rate and staining intensity: 0~3 as a weak signal; 4~7 as a strong signal.

**Immunohistochemistry (IHC) analysis**

For frozen sections, samples were returned to room temperature for 0.5 h and then rehydrated. For paraffin sections, samples were dewaxed and rehydrated. Endogenous peroxidase activity was blocked with hydrogen peroxide. The slides were placed in citric acid buffer for antigen recovery. Nonspecific immunoglobulin binding was blocked by incubation with 5% albumin bovine (AMRESCO, USA) at room temperature for 30 min. After an overnight incubation with primary antibody at 4 °C, the slides were then incubated in HRP-labeled polymer, according to the manufacturer’s instructions stained with DAB (ZSGB-BIO, China) and then co-stained with hematoxylin to visualize nuclei.

**Plasmid construction, transfection and lentiviral production**

Plasmids were transfected into cells using Lipofectamine 2000 (Invitrogen, Carlsbad, CA). The lentivirus was packaged by co-transfecting 293FT cells with the recombinant lentiviral transfer plasmid, packaging (p8.9) plasmids, and envelope (VSV‐G) plasmid using the lentiviral packaging system (Jiman Biotech, Shanghai, China). 18 h after transfection, the medium was changed, and cells were cultured for additional 48 h. Then the supernatant containing the virus was harvested. The lentivirus packaged with the empty pLVX-ACGFP-N1 vector served as a control.

**Colony formation and cell viability assay**

For colony formation assay, TNBC cells were seeded in a 24-well plate at a density of 500 cells/well, and treated with or without 1 μM AM580. After incubation for 14 days, colonies were stained with crystal violet for 15 min and counted. The MTT assay was used to determine cell viability. Cells were seeded on 96-well plates at 3000 cells/well overnight, and treated with different concentrations of RARα agonist for indicated times. 20 μL MTT solution (5 mg/ml) was added to each well at the end of the treatment, and incubated for another 4 h. Formed formazan crystals were dissolved in 100 μL DMSO and the absorbance was measured at 570 nm on a microplate reader (Bio-Tek, CA, USA). IC_50_ value was calculated by GraphPad Prism 5.0 software (CA, USA).

**Western blotting (WB) analysis**

Cells were harvested and lysed on ice in RIPA buffer supplemented with phosphatase inhibitors PMSF and protease inhibitors aprotinin. The protein concentration was determined by BCA protein concentration assay kit (Beyotime, ShangHai, China). Equal quantity of proteins was then separated on SDS-PAGE gels and transferred to PVDF membrane. The membranes were then blocked with 5% nonfat milk at room temperature for 1 h and incubated with primary antibodies overnight at 4°C. Next, the membranes were washed three times with TBS-T (Tris-buffered saline-5% Tween 20) and incubated with the HRP-conjugated secondary antibody for 2 h at room temperature. Chemiluminescent detection was performed by ECL (BIO-RAD, USA). β-actin, GAPDH, or β-Tubulin served as a loading control.

**Cell cycle analysis**

Cell cycle analysis was performed using PI/RNase staining and flow cytometry. Briefly, 5×10^5^ cells were seeded in 6-well plates, and treated with or without AM580 for 48 h. Cells were then washed with ice-cold PBS and fixed in 75% ethanol at -20°C overnight. Cells were again washed with PBS and then incubated with PI/RNase (0.5 ml/test, 1 × 10^6^ cells) in dark at 37 ℃ for 15 min. Samples were analyzed by Guava Easy Cytometer and DNA content was quantified using Modfit software (Verity Software House, USA).

**Detection of cell apoptosis**

Quantification of apoptotic cells was performed using the Annexin V-APC/7-AAD Apoptosis Detection Kit (Becton Dickinson) by flow cytometry. Briefly, 5×10^5^ cells were seeded in 6-well plates and treated with or without AM580 for 48 h. Then cells were harvested, stained with Annexin V-APC/7-AAD and analyzed by Guava Easy Cytometer (Guava Technologies, Merck Drugs & Biotechnology, Germany).

**Acridine orange (AO) staining**

Cells were seeded in 6-well plates and treated with AM580 for 72h. Cells were then washed twice with ice-cold PBS, stained with 1 µg/mL AO for 15 min, and observed under a fluorescence microscope (Nikon, Tokyo, Japan).

**RNA isolation and quantitative reverse-transcriptase PCR (qRT-PCR)**

Total RNA was extracted using Trizol agent (Invitrogen, USA). qRT-PCR was performed in triplicate. Primers were listed as follows: RARβ_2_: 5’-TTCAAGCAAGCCTCACATGTTTCCA C-3’ (forward), 5’- AGGTAATTACACGCTCTGCACCTTTAG-3’ (reverse). Caspase-9: 5’-CTCAGACCAGAGATTCGCAAAC-3’ (forward), 5’-GCATTTCCCCTCAAACTCTCAA-3’ (reverse). p21: 5’-CGATGGAACTTCGACTTTGTCA-3’ (forward), 5’- GCACAAGGGTACAAGACAGTG-3’ (reverse). C/EBPε: 5’-CTCCGATCTCTTTGCCGTGAA-3’ (forward), 5’-CCGAAGGTATGTGGAGGGTAG-3’ (reverse). CYP26A1: 5’-ATGTTCCGAATCGCCATGC-3’ (forward), 5’-GAGAAGAGATTGCGGGTCATTT-3’ (reverse). DHRS3: 5’-ACTGAGTGCCATTACTTCATCTG-3’ (forward), 5’-CATCACTGTCCATTAGGCTCTTC-3’ (reverse). miR-3074-5p: 5’-CGCGGTTCCTGCTGAACTG-3’ (forward), 5’-AGTGCAGGGTCCGAGGTATT-3’ (reverse). GAPDH: 5’-AACCCTTAAGAGGGATGCTGC-3’ (forward), 5’-ATGAAGGGGTCGTTGATGGC-3’ (reverse). U6: 5’-CTCGCTTCGGCAGCACA-3 (forward), 5’-AACGCTTCACGAATTTGCGT-3’(reverse). GAPDH and U6 was used as an internal control of RNA integrity. The relative expression levels of miRNA were determined by qRT-PCR using Mir-X™ miRNA First-Strand Synthesis kit (Takara, DaLian, China), according to the manufacturer's protocol. An Applied Biosystems® 7500 HT Fast Real-Time PCR System was used and the reaction conditions were as follows: 95°C for 10 sec, 40 cycles at 95°C for 5 sec and 60°C for 20 sec. For the PCR reaction, 2 µl cDNA solution, 13 µl PCR master mix, 1 µl of primers and 9 µl H_2_O were mixed to obtain a final reaction volume of 25 µl. The mRNA expression levels of DHRS3 was detected by qRT-PCR using the standard SYBR-Green RT-PCR kit (Takara, DaLian, China), according to the manufacturer's protocol. The relative expression levels were quantified using GraphPad Prism 5.0 software (GraphPad Software, Inc., San Diego, CA, USA), and the 2-ΔΔCq method.
